# Role of birth order, gender, and region in educational attainment in Pakistan

**DOI:** 10.1038/s41598-022-15700-x

**Published:** 2022-07-12

**Authors:** Syed Hassan Raza, Zulfiqar Ali Shah, Wajiha Haq

**Affiliations:** 1 School of Economics, Quaid-I-Azam University, Islamabad, Pakistan; 2 School of Social Sciences and Humanities, National University of Science and Technology, Islamabad, Pakistan

**Keywords:** Psychology and behaviour, Socioeconomic scenarios, Health care economics

## Abstract

We studied the effects of birth order and socioeconomic factors on educational attainment in Pakistan. We examined this relationship by using PSLM/HIES 2018–19 which is nationally representative household survey data. We found striking evidence that being born first as a male child is positively and significantly associated with educational attainment. Whereas in our girls' sample we found that being born first is significantly and negatively associated with educational attainment, and this effect does not persist for second-born female children if the firstborn is a male child. Such a difference in our estimates led us to investigate further the cohort and rural–urban dimensions. We concluded that urban areas in Pakistan are primarily responsible for resource rationing in favor of male children for younger cohorts. Therefore, the study recommends the targeted policy intervention to remove such differentials based on gender when it comes to the educational attainment of a child.

## Introduction

It is widely acknowledged that education affects the economic conditions of people by increasing their earnings in the labor market^1^. Education helps people to learn different skills which in turn facilitates them to get employment and access to opportunities^2^. Therefore, the higher the educational attainment, the higher will be the possibility to get better employment along with higher wages, and higher wage growth. Hence, it could be argued that educated people are more productive which makes them less vulnerable to unemployment. In addition to the idiosyncratic benefits of education, it affects a nation’s progress considerably. Countries with better human capital are less vulnerable to poverty and income inequality. Countries equipped with better human capital have various other benefits such as chances of good governance, social inclusion, and freedom from social problems like crime rate, etc^3^.These educational benefits can be ascertained through an egalitarian educational policy which can dampen the regional disparities and ensure inclusiveness. This paper explores the hindering factors to education concerning birth-order, gender in rural and urban areas and recommends policies that ensure gender inclusion.

Education, which researchers have objectified as educational attainment, depends upon the education policy of a country, mode of the labor market, preferences of the people in a country, and various other national factors. Apart from national factors educational attainment also depends upon household characteristics such as socio-economic conditions, demographicfactors, cultural preferences etc. Moreover, Idiosyncratic characteristics do also play a prominent role in the educational attainment of individuals such as sibling size, gender, intelligence, and more importantly birth order. Recent literature in economics has validated the importance of birth order while studying the educational outcomes^4–7^. The importance of birth order can also be ascertained from the fact that it has an intertemporal effect, for instance, those who are first born, not only their educational attainment will be higher but the attainment of their offspring will also be higher^8^.The cultural preferences for different genders, especially in orthodox cultures can be understood through birth order, where parents intend to produce more children if the firstborn is a girl^9^. In such societies sons provide more support to their parents and therefore they are paid much attention when it comes to education. To understand the theoretical framework and linkages through which birth order creates an impact on educational attainment, it is pivotal to understand the resource dilution hypothesis and other empirical work at the cross-section of the above-mentioned ideas. First, the resource dilution hypothesis which posits that the subsequent number of children decreases the number of parental resources at their disposal Parents provide various finite resources to their children against which they are competing with each other; parents can provide basic needs of life, cultural goods, and environment, in addition to the opportunities to engage with the outside world. Therefore, it could be argued that they provide personal attention, schooling, and treatment. But with each additional child the parental resources available to a particular child decrease, thus, hindering the chances of higher educational attainment for later born. The earlier born has advantages because one does not have to share resources with other children. Such parental resources are crucial for better educational outcomes of earlier born and vice versa. Regardless of whether we accept guardians who disseminate assets similarly among their kids, earlier-born are benefited more than later-born children^10^. Second, the Confluence hypothesis (Confluence model claims that the intellectual development within a family depends on cumulative intellectual environment where a first born enjoys rich cognitive stimulation as compared to latter born children) posits that the first born directly interacts with cognitively mature parents thus enhancing his chances of intelligence as compared to later born children who acquire a diluted intellectual environment^11^.

To understand the resource dilution model and birth order effects it is pertinent to incorporate family size because a large family size is associated with a large number of children thus the birth order effects are more apparent for large families. The existing literature to date has shown that family size is inversely associated with educational attainment^12,13^.

The resource dilution model recommends us to take into consideration various other socio-economic conditions and idiosyncratic characteristics such as household income (family income has shown consistent and positive impact on education^14^), family structure, gender of an individual, education of parents, and demography to better understand the role of birth order on educational attainment. Parental education cannot be relegated while studying the educational outcomes because empirical literature suggests it can determine the educational attainment of children (children born to educated parents are more educated as compared to children who belongs to uneducated parents^15^). In addition, household assets do also play a positive role in educational attainment for males while it is inversely related to females^16^. If Parental time spent with children is assumed more valuable for children, then firstborn children are at an advantage since they spend more time with parents as compared to other siblings during their infancy^17^.This earlier time investment coupled with other birth order effects put the firstborn child in an advantageous position which helps the one to attain higher education. Parents usually show firm attitudes to the firstborn to deter the latter born from the careless attitude towards studies. These strategies, they deem, are the defining factors that help the earlier to excel in academics^18^.

Other than the educational outcomes birth order has far-reaching impact on an individual’s life. For example, Birth order and cognitive abilities are strongly correlated with each other. It has been shown that the first and only children perform better in reading and they had higher educational aspirations than latter-born children^19^. These results are true for higher-income families only. Likewise, other social outcomes of an individual’s life are also interdependent on birth order such as occupation, health, and personality trait^20–22^.

The education system in Pakistan is managed by the federal ministry of education and provincial governments where the federal government is responsible to develop curricula, financing research and development, and accreditation. Pakistan shares a diverse educational system with differently molded serving institutes such as private, public, profit, and non-profit institutes. As for 260,803 registered institutes that are currently serving in Pakistan, 41,025,645 individuals are receiving education out of which 70% are public institutes and 30% are private^23^. This cannot even make the 70% of the population which is 212.75 million. According to UNICEF research, 22.8 million children aged 15–16 are not going to school representing 44% of the total population of this age group. They are still the victims of child labor, street crimes, and many other social evils. The literacy rate varies from central to peripheral areas such as 82% for Islamabad while it is 23% in the Torghar District. Furthermore, it varies by gender regionally, for example, the female Literacy rate in tribal areas resides in 9.5%, while it is 74% in Azad Jammu & Kashmir. Pakistan has a low literacy rate and has the second-largest out-of-school population (16.8 million children) after Nigeria.

The lower educational attainment in Pakistan is subject to various causes. First, a rapidly growing population coupled with economic contraction does not provide people access to smooth employment which in turn lessens the possibilities for parents to get their children an education. Second, expanding middle-income households in Pakistan paints a problematic picture where there are fewer earners and more dependents which makes it hard to afford education for every individual. Third, complex familial structure along with cultural preferences provides skewed opportunities to people based on gender, where boys owing to higher economic benefits attain higher and better education as compared to girls. Therefore, it could be argued that in a developing country like Pakistan where parents have to ration the resource to a gender which can potentially bring more returns.

Like other developing countries people in Pakistan resides in a different familial set-up which evidently predicts their later outcomes in life^24^.To incorporate the effects of familial set-up on adult outcomes it is pertinent to include the role of birth order as it provides a reliable instrument to household size. The skewed educational opportunities in Pakistan can also be understood through birth order, as it has been witnessed that people prefer to produce more children if the first born child is female^25^.The literacy rate in Pakistan is 64.7% where the majority of the adult population are uneducated or less educated^26^, who prefer to produce a large number of children, particularly sons, to attain better economic returns^27^, thus through birth order of children we can easily learn about parents idiosyncratic characteristics such as education, wealth, demographic belonging, etc.

A major part of the research has been done for different countries but to the best of our knowledge, no work has been done to exploit the role of birth order in educational attainment in Pakistan. This study will be the first of its kind to help understand the role of birth order in the educational attainment of first born male and female children in Pakistan. In this paper, we thoroughly examine the relationship between birth order and educational attainment for nationwide data followed by a regional analysis. Lastly, we incorporate the cohort analysis to study whether the birth order effects are recent or old phenomena. Based on the findings the study aims to draw policy recommendations to improve educational attainment in Pakistan.

## Data

Pakistan Bureau of Statistics regularly surveys to provide detailed information about the characteristics of household members. Since 1963 the Household Integrated economic survey has been conducted at every alternate year. During the early 1990s, it revised its Questionnaires to meet the requirements of the National account. Moreover, in 1999 its data collection method and questionnaire were revised again to reflect the integration of the Pakistan Integrated Household Survey (PIHS). Later on, it has been renamed Pakistan Social and Living Measurement (PSLM). This paper has used PSLM 2018–19 microdata, which is the eleventh round of a series of surveys. PSLM 2018–19 provides detailed information about 24,809 households comprising 1802 urban and rural sampling units. The HIES provides detailed information on outcome indicators on Health, Housing, education population and welfare, hygiene, water sanitation, information communication and technology (ICT), food insecurity (FIES), and income and expenditure.

### Data availability

The data used for this current study is publicly available and can be accessed through the website of the Pakistan Bureau of Statistics repository (https://www.pbs.gov.pk/content/pslm-hies-2018-19-provincial-level-survey).

### Stylized facts

The HIES questionnaire asks every household member about their highest *“years of schooling.”* The data set provides ranges from Montessori up to Ph.D. Every numeral shows years of schooling completed and 17 shows M.s, M. Phil, and Ph.D., cumulatively. *“Birth order”* shows the ordering of children, where 1 shows firstborn and 0 shows later born. We have generated birth orders by counting the household members concerning their years of birth.

We have used real monthly income as a flow variable while the total wealth, comprising agriculture and non-agriculture wealth as a stock variable. The variable *“bad accommodation”* shows the absence of clean water, internet, and waste management while *“worried for food”* shows the household residents were worried for their food at some point due to lack of financial resources. We had total observations of 184,764 individuals but we restrict our analysis to those individuals who are above the age of education attainment i.e., 22 years old. Furthermore, we exclude those individuals who are currently studying. After restricting our data, we ended up with 8,729 sons and 2,679 daughters who fall under the above criteria. Table 1 shows descriptive statistics for sons and daughters. We divided our sample into three categories each for sons and daughters. First, we studied nationwide followed up by regional division and cohort analysis.

**Table 1:** Descriptive statistics sons and daughters.

Fortunate children of Pakistan: Role of birth order on education attainment		
Variable	Mean	SD
**Male (sons)**		
Years of education	7.19	5.06
Age	28.45	5.96
First born	.64	.47
Family size	3.97	2.02
Household income	25,026.82	28,528.5
Household wealth	2818.37	20,463.3
Bad accommodation	.82	.38
Worried for food	.11	.32
Observations	8729	
**Female (daughters)**		
Years of education	7.02	5.93
Age	26.86	5.57
First born	.80	.39
Family size	3.46	2.71
Household income	3976.7	11,384.1
Household wealth	4192.65	28,926.26
Bad accommodation	.79	.40
Worried for food	.12	.32
Observations	2679	

This table reports descriptive statistics (mean and s.d.) for sons (top panel) and daughters (bottom panel). Our main sample includes children aged above 22 years.

**Figure 1:**
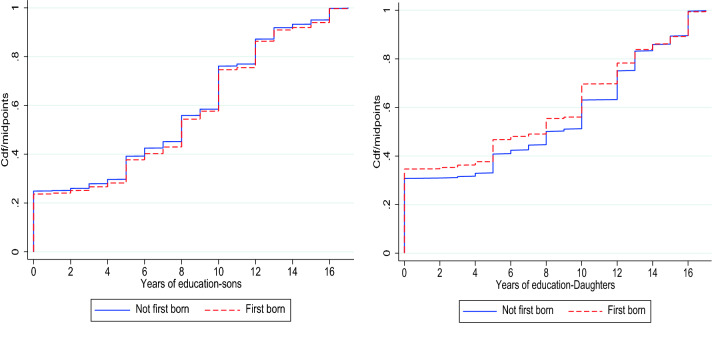
Distribution of years of schooling of sons and daughters by birth order. The first panel shows the relationship between cumulative distributions of education (Y axis) of sons by birth order and years of schooling (X axis). The second panel shows the relationship for daughters.

On average sons have 7.19 years of education and they were 28 years old at the time of the interview. Out of 8729 sons, 64% are firstborn in a family with an average number of children of 3.97. while daughters have on average 7.02 years of education and they were on average 26 years old at the time of the interview. Out of 2679 daughters, 80% of them are firstborn in a family with an average number of sibling of 3.46. Our data has primarily exploited the relationship between birth order and educational attainment of sons and daughters while incorporating other controlled variables. Figure 1 depicts the association between years of schooling of daughters and sons with cumulative distribution function by birth order.

**Figure 2:**
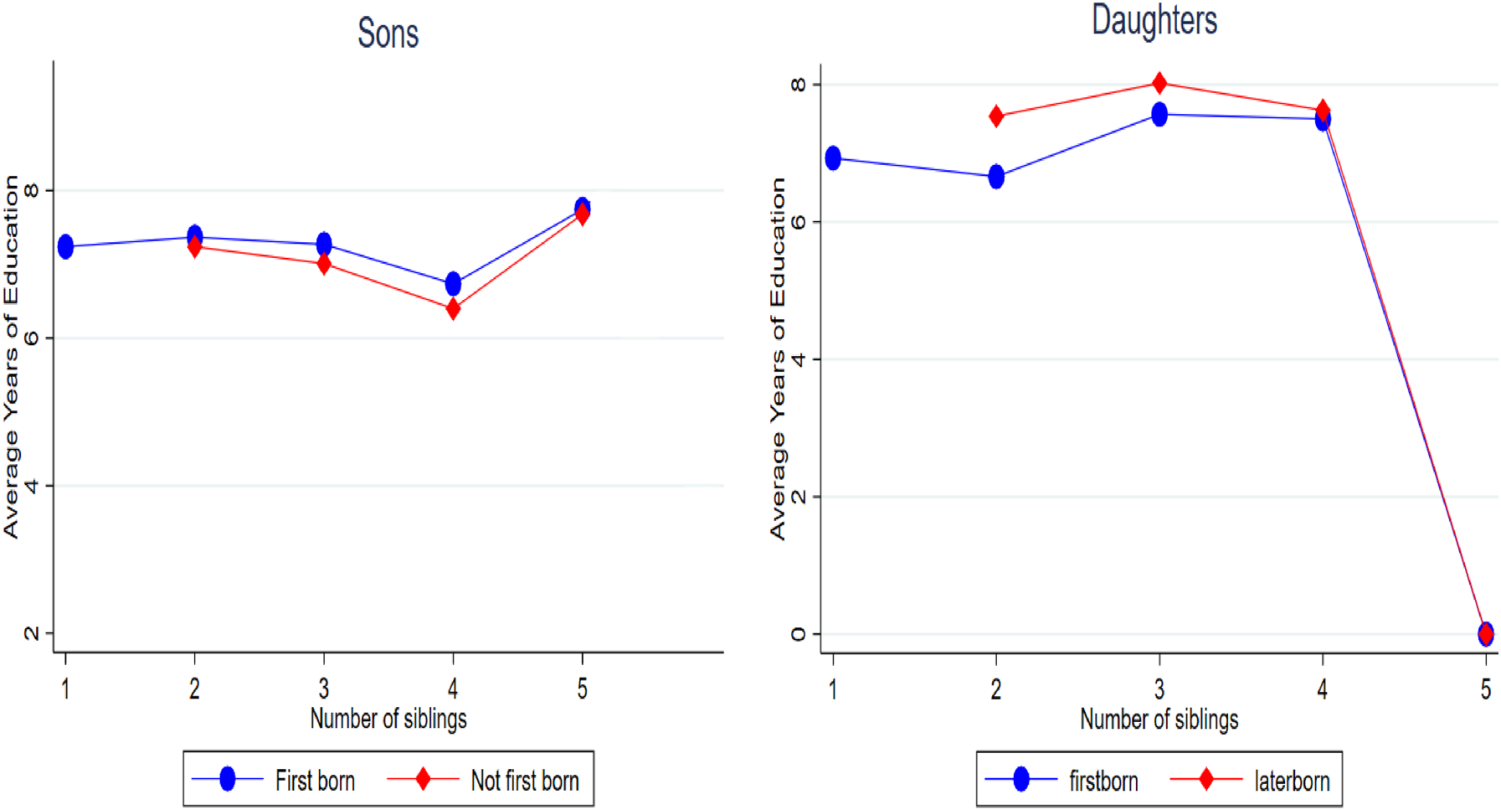
Average years of education for sons and daughters by birth order across the family sizes.

Figure 1 shows that the probability to lower years of schooling is greater for later-born children than first-born children. Though the effects are not that clear for sons but these effects are more pronounced for Daughters. For example, for daughters, the probability of a lower 10 years of education is 45 percentage points for later-born daughters while it is 55 percentage points for first born daughters. In Fig. 2 we plot the average years of education of sons and daughters by birth order controlling for family size. From the left-hand panel of Fig. 2, it is obvious that the average years of schooling for first-born sons are higher as compared to later-born sons across the different family sizes (number of siblings). From the right-hand panel of Fig. 2, it is clear that the latter-born daughters have higher average years of education as compared to first-born daughters. This relationship persists for a family size of 3 but thereafter we observe a convergence between the average years of schooling for both first-born and later-born daughters.

## Analytical framework

Resource dilution provides a suitable model to understand the role of birth order on education attainment using the within-family approach. As the parental resources are finite in specific households, therefore, we would incorporate other variables for parental resources as well such as household income, household wealth, etc. Our baseline model, establishes the association between the birth order of a child and its educational attainment (years of schooling).

1 $$Y{j}_{d}^{s}={\pi }_{0}+{\pi }_{1}\mathrm{C}{j}_{d}^{s}+{\pi }_{2}\mathrm{X}{j}_{d}^{s}+{\pi }_{3}\mathrm{M}{j}_{d}^{s}+{\psi }_{j}$$

where “y_j_^s/d^” stands for years of schooling of son or daughter born in order “j”, which is “1” for first born while it is “0” for later born. C_j_ represents the birth order of the child (either son or daughter). X_j_^s/d^ is the vector that represents a household’s socio-economic characteristics like household income, household wealth, and household size while M_j_^s/d^ is also a vector that represents household dwelling characteristics like proper waste management system, access to clean drinking water, access to an internet facility and worried for food. Where π_0,_ π_1_ and π_3_ show relevant parameters.

We have estimated Eq. 1 using the ordinary least square (OLS) technique since our dependent variable is a continuous one. First, we have estimated Eq. 1 on a nationwide sample followed by cohort analysis and regional analysis. We have employed only those individuals who are above the age of education attainment i.e., 22 years old.

## Empirical results

We extract the main results from Sect. 3.5 described in the empirical analysis. Table 2 shows the OLS regression results for daughters across nationwide, region, and age-cohorts. The coefficient for first-born girls is negative and statistically significant across columns 1, 3, and 5. Column 1 shows the main estimation nationwide with all other control variables e.g., family size, household income, household wealth (agricultural and financial), bad accommodation (households who lack clean drinking water, lack of internet facility with no proper waste management system), and worried for food (worried for food due to lack of financial resources). The family size is inversely related to the educational attainment of girls where the value of the coefficient in column 1 is “0.769,” followed by “0.789” in column 3 and “0.991” in column 5 with statistically significant results. Household income increases the educational attainment of girls by 1.143 years in column 1, “1.171” in column 3, and “1.056” in column 5 with statistically significant results 1% level. Those girls who are living in a family with greater wealth have higher chances of educational attainment. The coefficient for household wealth is “0.337” in column 1, “0.292” in column 3, and “0.359” in column 5 and are statistically significant. Bad accommodated households (households with no proper waste management system, unavailability of the internet, and those who lack clean drinking water) decrease the educational attainment of girls by “4.424” years in column 1, 4.288 years in column 3, and 3.341 years in column 5. The coefficient for bad accommodation is statistically significant at a 1% level.

**Table 2:** The effect of female(daughter) birth order on own education attainment.

Female years of schooling					
	Nation-wide	1st cohort (1952–75)	2nd cohort (1976–97)	Rural	Urban
First born	−0.769**	−0.148	−0.789**	−0.566	−0.991**
	(0.332)	(2.126)	(0.338)	(0.448)	(0.487)
Family size	−0.540**	0.618	−0.592**	−0.389	−0.682***
	(0.184)	(0.819)	(0.188)	(0.241)	(0.281)
Household income	1.143***	1.033**	1.171***	1.127***	1.056***
	(0.080)	(0.450)	(0.081)	(0.118)	(0.110)
Household wealth	0.337***	1.878***	0.292**	0.393**	0.359**
	(0.102)	(0.562)	(0.103)	(0.127)	(0.168)
Bad accommodation	−4.424***	−10.054***	−4.288***	−3.458***	−3.341***
	(0.256)	(1.816)	(0.259)	(0.949)	(0.309)
Worried for food	−3.317***	−3.062	−3.312***	−3.173***	−3.411***
	(0.312)	(1.850)	0.315)	(0.381)	(0.535)
Constant	9.889***	8.572***	9.937***	7.942***	10.541***
	(0.573)	(3.176)	(0.583)	(1.187)	(0.801)
N	2656	1166	1490	1500	1156
R2-adj	0.22	0.41	0.22	0.11	0.20

Robust standard errors are shown in parenthesis *p < 0.10, **p < 0.05, *** < 0.01.

Girls living in a family who are worried about food due to lack of financial or other resources have lower chances of educational attainment. The coefficient for worried for food is negative and its values are “3.317” in column 1, “3.173” in column 3, and “3.411” in column 5 and are statistically significant at a 1% level.

Table 3 shows the birth order effects on educational attainment for males (sons). The results reveal that for son’s birth order does play a positive and statistically significant role in educational attainment. The first-born sons have higher chances of education attainment of “0.261” years in column 1, “0.294” years in column 3 and “0.469” years in column 5. The coefficients are statistically significant at a 5% level across all the three columns. Family size is negatively associated with the educational attainment of sons with a coefficient of “0.621” in column 1, “0.066” in column 3, and “0.575” in column 5 and are statistically significant at a 1% level. The coefficients of household wealth are statistically significant at a 1% level with positive values of “0.381” in column 1, “0.380” in column 3, and “0.540” in column 5. Boys living in bad accommodated houses have lower chances of educational attainment. The value of coefficients for bad accommodation are negative having values “3.152” in column 1, “3.147” in column 3, and “2.833” in column 5, these coefficients are statistically significant at a 1% level. Boys dwelling in those households who are worried about food due to unavailability of resources have lower educational attainment with a coefficient value of “2.27” in column1, “2.243” in column 3, and “1.970” in column 5. These coefficients are statistically significant at the 1% level. The effects of birth order in a rural area are insignificant depicting that birth order does not matter evidently for education attainment in such areas. Column 4 provides information about the birth order effects in rural areas. Furthermore, the effects of birth order are not obvious for those people who were born between 1954 and 1976.

**Table 3:** The effect of male(son) birth order on own education attainment.

Male years of schooling					
	Nation-wide	1st cohort (1952–75)	2nd cohort (1976–97)	Rural	Urban
First born	0.261**	−1.416	0.294**	0.078	0.469***
	(0.125)	(0.993)	(0.129)	(0.173)	(0.186)
Family size	−0.621***	−0.480	−0.066***	−0.646***	−0.575***
	(0.064)	(0.294)	(0.066)	(0.086)	(0.096)
Household income	0.936***	1.859***	0.917***	1.096***	0.625***
	(0.052)	(0.269)	(0.053)	(0.070)	(0.078)
Household wealth	0.381***	0.539**	0.380***	0.362***	0.540***
	(0.052)	(0.270)	(0.053)	(0.063)	(0.096)
Bad accommodation	−3.152***	−3.085***	−3.147***	−2.728***	−2.833***
	(0.134)	(0.709)	(0.136)	(0.491)	(0.157)
Worried for food	−2.27***	−2.557**	−2.243***	−2.282***	−1.970***
	(0.158)	(0.819)	(0.161)	(0.189)	(0.293)
Constant	8.264***	6.015***	8.312***	7.456***	8.811***
	(0.251)	(1.590)	(0.255)	(0.569)	(0.355)
N	8661	2268	6393	5059	3602
R2-adj	0.15	0.30	0.14	0.09	0.14

Robust standard errors are shown in parenthesis *p < 0.10, **p < 0.05, *** < 0.01.

## Discussion

It is evident from our analysis that in Pakistan being a first-born girl is negatively associated with educational attainment. This implies when there is a first-born female child parents are more likely to keep the resource for the later-born child which they hope to be a male child. It is further evident from the robustness check we provided in Table 6 which states that if the first born is a male child and the second born is a female child her educational attainment will not be affected because she is born after a male child. This may be due to the fact, that spending on education in developing countries like Pakistan is considered to be an investment and parents tends to invest on a gender which can potentially bring higher return which is substantially low in case of females in Pakistan In addition to this, the possible explanations could be women are perceived to be unproductive and politically disempowered. The patriarchal norms have restricted women to the household where they are supposed to do unpaid work i.e., childbearing, taking care of children and other household members, and housework. Due to these reasons, there is a possibility that parents are reluctant to invest in the education of girls. At large, the girls in Pakistan are supposed to help their mothers with household chores and the first-born girl is usually seen as a second mother. She takes responsibility to take care of other family members in the absence of her mother. Therefore, it is right to imply that in Pakistan, the firstborn girl is not encouraged to get an education but she is trained to help out her mother at the earliest. The cultural and economic advantages to males are also contributing to the lower educational attainment of girls where people tend to prefer sons over daughters, thus, it could be argued that if the firstborn is a girl, it is predictive of large family size, parents produce more children to acquire son. The large family size coupled with cultural preferences leaves the girls with more diluted resources thus hampering the possibilities of higher education attainment of girls. The labor market in Pakistan provides unequal economic opportunities to female which decreases the economic importance of girls; the labor market in Pakistan offer low-wage jobs to women; deny facilities that women biology demands, thus disincentivizing parents to invest in girl’s education^28^.In addition to this, in Pakistan parents invest in their children’s education to get earlier returns since girls cannot give returns back to their parents, they would more possibly give returns to their in-laws (due to early marriages) which causes parents to oppose the investment to girl’s education^29^. Our cohort analysis explains the fact that in the older generation (i-e those who were born between 1952 and 1975) the birth order for girls played an insignificant and negative role, which shows that since women were largely uneducated thus the effects of birth order on education were trivial. With familial and economic restructuring, the birth order is playing a prominent role among those girls who were born between 1976 and 1998. It is noteworthy here that during this period Pakistan underwent remarkable economic and political restructuring which affected the social fabric of the society. Since families living in the rural area belongs to joint and extended families where, arguably, a girl has no say in education attainment thus shows that the role of birth order is peripheral in rural areas while birth order shows a considerable role in an urban area where a first-born girl is at disadvantage.

The results from our son’s sample show that being firstborn has higher chances of educational attainment depicting that parents do invest in the education of firstborn considerably to get returns early or higher returns. Because in Pakistan, like many other developing countries, males are perceived to be the breadwinner for the family, therefore, a male is supposed to do work to meet the expenses of his family, thus, parents prefer to invest in the education of sons and particularly the firstborn. These effects are more pronounced in urban than in rural areas depicting that the educational attainment in the rural area is lower where sons are following the footsteps of their fathers to look after the familial or traditional wealth and in the extended family system. While in an urban area the family configuration demands the firstborn to get an education to help his father to meet his family expenses. The effects across the age cohorts reveal that people born before 1976 have no significant effects showing that education attainment solely depends upon opportunities available to a particular child or the factors which one can potentially control, unlike birth order. Those children who were born between 1976 and 1997 provide strong evidence for birth order which shows changes in the family configuration where a firstborn child is preferred over the latter-born child.

The family size shows similar effects for girls and boys: education attainment decreases with an additional increase in family size. Household income is positively associated with education attainment with stronger effects for females than males showing that those families who have smooth income tend to prefer for their daughters more education. Moreover, household wealth is also positively associated with the educational attainment of girls and boys with stronger effects for boys. The different impacts of income and wealth are interesting findings, where those households who inherit family wealth and inheritance, tend to maintain the legacy by empowering their sons, while those parents who earn a livelihood through employment considers girls' education attainment of vital importance. It is important to note that girls living in such households which do not have access to clean drinking water, internet facilities and proper waste management systems have a detrimental effect on the girl’s education in comparison to the boys. It could be that girls are responsible to manage these deprivations such as fetching clean drinking water, managing waste, etc. Our findings ascertained that the education of girls suffers deeply in those households which are food-deprived or worried for food in comparison to the boys. Therefore, we imply that male education is associated with better and early economic yields.

## Conclusion

Though economic literature provides ample evidence for the negative association between birth order and educational attainment across different countries. But no work is evident in the context of Pakistan. Since Pakistan is a country where the educational attainment of a child depends solely on the socio-economic status of a household, where parents distribute resources to their children based on their potential possibilities for earlier and higher economic returns in addition to some of the factors which are beyond their control such as birth order. This study tries to fill the literature gap by exploiting the role of birth order on education for sons and daughters. The study also analyzes the birth order effects for age cohorts with rural and urban breakdown. The study relies on Household Integrated Economic Survey (HIES) 2018–2019 with a sample of 8681observation for sons and 2656 observations for daughters. We observed that being firstborn increases the chances of educational attainment of sons by 0.261 years but decreases the education attainment of girls by 0.769 years with. The results are statistically significant at 5% level. Our findings explain the deep embedded cultural preferences for males owing to the economic advantages associated with them. Our findings across the rural and urban areas show that in urban areas being firstborn is associated with higher education attainment for boys while negative for girls while in rural areas the effects are insignificant. Furthermore, the results across the age cohorts show that before 1976 birth order did not play a significant role while after 1976 firstborn people were given preferred and they have reportedly higher education. Furthermore, it is evident from our analysis that household income and household wealth are the factors that plays a prominent role across all the analysis. It can be understood that the role of income and wealth are necessities to attain higher education. This study also provides evidence for the role of dwelling characteristics of a household in the attainment of higher education of a child. It can be seen from our analysis that children belong to those households where there is no adequate food, a lack of proper waste management system and a lack of other basic amenities to education have lower educational attainment than children who belong to better dwelling characteristics. Based on the above finding it can be argued that in those households’ income and wealth are insufficient to fulfill the basic amenities of households thus the spending wealth and income on the education of children is peripheral in nature.

### Policy recommendation

After analyzing HIES 2018–19 microdata we have come to certain conclusions which can be instrumental to help policymakers to make policies that help people to achieve higher education regardless of the factors beyond their control such as birth order. Our results reveal that females are not given priority when it comes to education and the plausible reason is that women in Pakistan are perceived to be unproductive. They are assumed to be caretakers of households and due to lower educational returns of females' parents are not investing in girls’ education. To ensure the economic participation of women government must incentivize parents to invest in girls’ education. This can be done by enacting women-friendly policies. In addition, the labor market offers lower wages to women in Pakistan, on top of denying the biological demands of women due to which women are reluctant to attain higher education. The government can ensure these unjust policies of firms by enforcing laws and making firms accountable to state rules and regulations. Our data shows that those households who have better socio-economic status have children with greater and higher educational attainment. This is the indication that the social safety net should be diverted to poor families especially those living in urban areas to ensure a higher enrollment and attainment rate of education. The social safety net diverted to urban areas will offer promising results in terms of educational attainment.

## Appendix A

### Robustness checks

In order to check the consistency of our results we applied different robustness checks. We check the effects of birth order by varying family sizes, following Black et al. (2018). Though by doing so our sample size will decrease but we will be able to compare the birth order effects. Table 4 shows the birth order effects for different family sizes.

**Table 4:** the effects of birth order on the education of female and male, separate regression by family size.

	Family size = 3	Family size > 3
**Female**		
First born	−0.654**	−0.214**
	(0.260)	(0.097)
N	1536	1143
R2_adj	0.021	0.39
**Male**		
First born	0.681***	0.295**
	(0.150)	(0.130)
N	6412	2237
R2_adj	0.159	0.119

In the top panel of Table 4 we show that being firstborn girl decreases the education attainment but this effect monotonically increases with family size, implying that girls born in large families have higher chances to lower educational attainment. The bottom panel of Table 4 shows that being a first-born son increases his chances of higher education attainment but the effects monotonically decrease with family size, implying that a son born to large families has fewer possibilities to higher education attainment than a boy born to a small family. Second, we drop those children who are the only children of their parents. Then we perform an estimation for those by incorporating birth order for different household sizes.

The results from Table 5 shows consistency of results. Where a girl born to larger family has higher possibilities to lower education attainment while in case of males, boys born to larger families have lower chances to higher education attainment. Finally, we checked the birth effects by dropping those children who have more than one sibling for different age-cohorts. Our results showed consistent results with slight anomaly. These tests depicts that our baseline specification is correct and better explain the correlation between birth order of child and its completed years of schooling.

**Table 5:** Effects of birth order for those households who are having more than one child.

	Family size = 3	Family size > 3
**Female**		
First born	−0.821**	−0.318**
	(0.374)	(0.151)
N	694	240
R2_adj	0.30	0.40
**Male**		
First born	0.113**	0.322**
	(0.050)	(0.157)
N	2940	2237
R2_adj	0.17	0.13

Finally, we have also checked the birth order effects for those girls who are the second born conditional on first born is a male child, and our results prove the fact from the earlier analysis that the first-born girl is disadvantageous as compared to the first-born sons or later-born girls. Furthermore, this is in line with our discussion that parents tend to keep the resource for male child as a priority investment and if the son is born first the negative effect of birth order for a girl with educational attainment changes into a positive one. It is evident here that if the first born is a male child and second born is a female child, the educational attainment of a second born child becomes positive and significant with her birth order and this was not the case in our earlier analysis where we observe being born first as a female child negatively effects the educational attainment (Table 6).

**Table 6:** Effects of second born girls on education on educational attainment.

Second born (girl)	0.213***
	(0.033)
N	14,690
R2_adj	0.0003
